# The prevalence of elder abuse in institutional settings: a systematic review and meta-analysis

**DOI:** 10.1093/eurpub/cky093

**Published:** 2018-06-05

**Authors:** Yongjie Yon, Maria Ramiro-Gonzalez, Christopher R Mikton, Manfred Huber, Dinesh Sethi

**Affiliations:** 1 WHO Regional Office for Europe, Copenhagen DR-2100, Denmark; 2 WHO Headquarters, Geneva 1202, Switzerland

## Abstract

**Background:**

A recent study has shown that close to one in six older adults have experienced elder abuse in a community setting in the past year. It is thought that abuse in institutions is just as prevalent. Few systematic evidence of the scale of the problem exists in elder care facilities. The aim of this review is to conduct a systematic review and meta-analysis of the problem in institutional settings and to provide estimates of the prevalence of elder abuse in the past 12 months.

**Methods:**

Fourteen academic databases and other online platforms were systematically searched for studies on elder abuse. Additionally, 26 experts in the field were consulted to identify further studies. All studies were screened for inclusion criteria by two independent reviewers. Data were extracted, and meta-analysis was conducted. Self-reported data from older residents and staff were considered separately.

**Results:**

Nine studies met the inclusion criteria from an initial of 55 studies identified for review. Overall abuse estimates, based on staff reports, suggest that 64.2% of staff admitted to elder abuse in the past year. There were insufficient studies to calculate an overall prevalence estimate based on self-reported data from older residents. Prevalence estimates for abuse subtypes reported by older residents were highest for psychological abuse (33.4%), followed by physical (14.1%), financial (13.8%), neglect (11.6%), and sexual abuse (1.9%).

**Conclusions:**

The prevalence of elder abuse in institutions is high. Global action to improve surveillance and monitoring of institutional elder abuse is vital to inform policy action to prevent elder abuse.

## Introduction

Elder abuse is an important public health issue with serious social, economic and health consequences. The global prevalence of past year elder abuse in the community settings is 15.7%, or approximately one in six older adults.^1^ According to the World Health Organization (WHO), elder abuse is defined as ‘a single, or repeated act, or lack of appropriate action, occurring within any relationship where there is an expectation of trust which causes harm or distress to an older person’.^2^ Elder abuse can be categorized according to: type of abuse—psychological, physical, sexual, and financial abuse and neglect; type of abuser—family members, informal and formal caregiver, or acquaintance; or setting in which it occurs—in the community and in an institution.^3^ Within institutional settings, abuse can be broadly categorized into resident-to-resident abuse or staff-to-resident abuse.^4^

Compared with research on other forms of interpersonal violence, elder abuse research, especially in institutions, is still in its infancy.^5^ However, research has shown that elder abuse occurs in every country with nursing and residential facilities and anecdotal evidence suggests that abuse may be very prevalent.^6^ A survey of nursing home staff in the USA indicated that 40% of staff admitted to committing psychological abuse in the past year and 10% to committing physical abuse.^7^ A systematic review of institutional abuse indicated that physical abuse often occurs as a form of staff retaliation against physically aggressive residents.^8^ Similarly, staff reported that they were more likely to withhold choices from aggressive residents.^9^ In another US national study, 1.5% of staff self-reported that they have committed theft.^10^ There is significant awareness of the issue of elder abuse in institutional settings among the population in European Union (EU) countries. According to a 2007 Eurobarometer special report on health and long-term care in the EU, 47% of European citizens think that poor treatment, neglect and abuse of older adults are common in their country.^11^

There is a gap in the current literature on the prevalence of elder abuse in nursing and residential facilities for older people. The need for greater attention to this topic stems from a number of factors. First, according to the data from *World Population Prospects*, in 2015, the global population of older adults aged 60 years or over is about 901 million or 12.3% of the world’s population, and by 2050, the global population of older adults will more than double to nearly 2.1 billion or 21.3%.^12^ Second, the number of ‘oldest-old’ adults, aged 80 years or over, is growing faster than the population of older adults. For example, by 2050, the number of the ‘oldest-old’ population will have more than tripled to 434 million from 125 million in 2015.^12^ Third, women, on average, have a longer life expectancy than men, and as a result they account for 61.6% of those over 80 years^12^. Fourth, it is likely that females and the ‘oldest-old’ seniors in the future will remain the largest age group in long-term care facilities.

Currently, older adults also make up the largest proportion of adult populations living in institutions for adults with mental disabilities in the European region.^13^ Residents of such facilities are more likely to have multiple forms of impairment including mental, physical or behavioural abnormalities as well as disabling conditions. Thus, due to their frailty, residents in institutional settings tend to be more dependent on others for care and may be at greater risk for abuse and neglect than older adults in community settings.^4^ Finally, the prevalence of abuse may be much higher than reported since under-reporting is estimated to be as high as 80%.^14^ Such under-reporting could be due to victims’ inability to communicate their abuse or due to their fear of repercussion and retaliation.

Urgent action is needed to prevent elder abuse from occurring, especially in the institutional settings. The WHO global strategy and action plan on ageing and health (2016–20)^15^ provides a roadmap to prevent elder abuse and achieve healthy ageing. The strategy calls for key actions in the areas of health systems, age-friendly environments, better long-term care and improvements in measurement, monitoring and research. Underlying this strategy is a set of core principles to ensure older adults age safely in a place that affirms their basic human rights and fundamental freedoms.^15^ Such affirmation is crucial to elder abuse prevention. Similarly, one of the supporting interventions in the WHO strategy and action plan for healthy ageing in Europe (2012–20) targets elder abuse prevention, which calls for actions to improve the quality of services within institutional settings.^16^ Despite increasing attention, research on institutional abuse is still lacking. To better capture and summarize existing research on institutional abuse, this systematic review and meta-analysis aims to synthesize prevalence estimates of abuse in institutional settings from existing literature and to identify gaps for future research directions.

## Methods

### Search strategy and selection criteria

This research, focused on institutional settings, was part of a larger systematic review of studies examining the prevalence of elder abuse in all settings. The study conforms to the Preferred Reporting Items for Systematic reviews and Meta-Analysis – or PRISMA – guidelines and has been registered with PROSPERO International Prospective Register of Systematic Reviews (CRD42015029197). A detailed description of the method has been published elsewhere.^17^ A brief description of the methodology is presented below.

A comprehensive four-step search strategy was used to identify relevant studies. The first step consisted of searching the following 14 academic databases from inception to 26 June 2015: PubMed, PsycINFO, CINAHL, EMBASE, MEDLINE, Sociological Abstracts, ERIC, AgeLine, Social Work Abstracts, International Bibliography of the Social Sciences, Social Services Abstracts, ProQuest Criminal Justice, ASSIA, Dissertations & Theses Full Text and Dissertations & Theses Global. A search strategy was developed for each database using a combination of free text and controlled vocabulary (i.e. MeSH terms). Additional search terms were included in consultation with an information specialist (librarian) who has extensive experience in systematic reviews. Some of the search terms used included: older adults, frail elderly, aged, elderly, seniors, elder abuse, elder neglect, elder mistreatment, elder maltreatment, domestic violence, intimate partner violence, abuse, violence, aggression, crimes, harmful behaviour, anger, rape, hostility, conflict, verbal abuse, physical abuse, sexual abuse, emotional abuse, prevalence, incidence, morbidity and epidemiology; nursing homes, assisted living, residential care institutions, residential facilities, health facilities and skilled nursing facilities. The full search strategy and search terms have been previously published.^1^^,^^17^

Second, reference lists of publications retrieved in the first step were screened for relevant studies. Third, we searched additional web-based platforms including specialized journals, Google for grey literature, and the WHO’s Global Health Library for scientific literature published in low and middle income countries. Finally, 26 experts in the field were consulted by e-mail, representing each of the six WHO regions (i.e. Africa, Americas, South-East Asia, European, Eastern Mediterranean and Western Pacific) to identify any studies that the first three steps may have missed up to 18 December 2015. Articles were independently screened in two stages by two reviewers: first, titles and abstracts were screened for relevance. This was followed by the retrieval and screening of full text articles by two reviewers using the eligibility criteria described below. If several publications reported on a single study, the publication that provided the most data were selected for further synthesis. Inter-rater reliability was analyzed using the Statistical Package for Social Sciences (SPSS Statistics 21). This analysis showed high levels of agreement between the reviewers (*κ*: 0.86–0.96). Disagreements were resolved through discussion, or with the help of a third reviewer.

Inclusion criteria were institutional-based samples that provided estimates of abuse prevalence at a national or sub-national level (e.g. states/provinces, counties, districts and large cities); and inclusion of participants that were 60 years of age and older, in line with a frequently used age limit used for data presentation and research.^18^ We excluded studies that were reviews, conference proceedings or used qualitative methods only, and studies that focused exclusively on use of restraints, self-neglect or homicide.

### Data extraction and quality assessment

Data were extracted by two reviewers: the first extracted data from the publications and the second cross-checked for accuracy. Three main categories of data were extracted: characteristics of the samples, methodological characteristics of each study and prevalence estimates of elder abuse and its sub-types. The study quality was assessed as part of the data extraction strategy by two reviewers using the Modified Newcastle-Ottawa Scale^19^ designed to assess the quality of non-randomized epidemiological research. To assess the risk of bias, reviewers rated each of the 7 items along a 4-point Likert scale from high risk of bias (i.e. 0) to low risk of bias (i.e. 3, see Panel 1). An overall score was calculated by adding all the items, thus, higher scores indicated lower risk of bias and stronger methodological quality.

### Data analysis

Meta-analysis was performed to synthesize the prevalence estimates. The decision to do a meta-analysis was made *a posteriori* after ensuring sufficient studies with similar characteristics were available for meta-analysis. Prevalence rates were calculated from raw proportions or percentages reported in the selected studies. The pooled estimates and the 95% confidence intervals (CIs) were calculated based on a random-effects model. Non-overlapping CIs were considered as an indication of statistical significant differences.^20^ All analyses were conducted using Comprehensive Meta-Analysis software (CMA 3.9).^21^ Heterogeneity tests with Higgins’ *I*^2^ statistic were performed to determine the extent of variation between the studies.^20^ Duval and Tweedie’s Trim and Fill method was performed to assess the degree of publication bias, its effect on the study findings, and to remove extreme outliers to correct for publication bias.^20^^,^^22^

## Results

**Figure 1:**
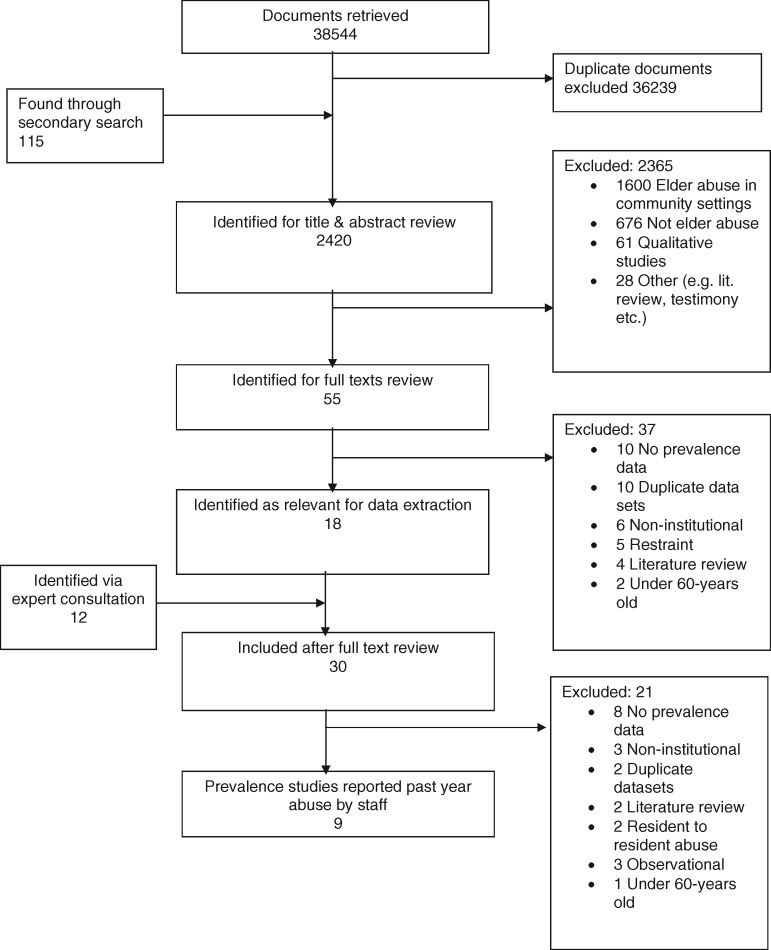
Summary of study selection for inclusion in analysis

Of the 38 544 studies that were initially identified through the comprehensive search strategy for all elder abuse prevalence studies occurring in the community and the institutional settings, 55 full-text articles related to abuse in the institutions were independently reviewed. These relevant articles fall into two categories of institutional abuse: resident-to-resident abuse and staff-to-resident abuse. From these, 18 studies were selected for data extraction and 12 additional studies were identified through expert consultations. After further screening, 21 studies were excluded and 9 studies were selected for meta-analysis, which provided data for staff-to-resident abuse. Among these, four studies^23–26^ examined abuse prevalence self-reported by older adults including one study in which abuse was reported by proxies, close relatives to the older adults^26^ and six studies in which abuse prevalence was self-reported by staff.^23^^,^^27–31^Figure 1 shows the flowchart of study selection.

The four prevalence studies, based on self-report by older adults and their proxies, were from the Czech Republic, Israel, Slovenia and the USA. In the studies, between 64.8 and 82.8% of the samples were women.^23^^,^^25^^,^^26^ Two studies provided age group breakdowns with those aged 75 years and older making up 75% of the samples.^23^^,^^25^ The older adults who participated in the studies were adults with normal cognitive functioning who had the ability to communicate and orient themselves in time and space. However, the majority of these respondents was frail and required assistance in activities of daily living (ADLs). The quality of the studies was assessed using the Modified Newcastle-Ottawa Scale.^19^ The maximum score for good quality study on this scale (i.e. low risk of bias) was 21. On average, the studies scored 11 out of 21 on the scale.

**Table 1:** Institutional abuse reported by older adults and staff

Elder abuse types	Pooled estimates (%)	Lower limit (%)	Upper limit (%)
**Reported by older adults over past year**			
Psychological (3 studies)	33.4	6.3	78.9
Physical^a^ (4 studies)	14.1	1.9	58.3
Sexual (3 studies)	1.9	0.03	59.2
Neglect (3 studies)	11.6	0.4	81.8
Financial (3 studies)	13.8	0.7	78.3
**Reported by staff over past year**			
Overall (4 studies)	64.2	53.3	73.9
Psychological^a^ (5 studies)	32.5	16.1	54.6
Physical^a^ (5 studies)	9.3	4.4	18.4
Sexual (3 studies)	0.7	0.04	11.7
Neglect^a^ (4 studies)	12.0	2.6	41.4

a Adjusted for publication bias.

There were six studies that were based on self-reports by staff. In these studies, staff were asked whether they had perpetrated or directed abusive acts to older residents. These studies were geographically diverse from the Czech Republic, Germany, Ireland, Israel and the USA. Of the six studies, between 80 and 97% of the staff respondents were women. All of the respondents were over 35 years old,^23^ with five studies reporting average staff ages of early- to mid-40 years old.^27–31^ There was a wide range in the average number of years of professional experience working with older adults: from less than 4 years^23^ to between 10.4 years^27^ and 13.8 years.^30^ Moreover, between 38 and 63% of the staff were registered nurses, licensed practical nurses or had received qualifications in the care of older adults.^28^^,^^29^^,^^31^ The characteristics of the older adults residing in the institutions were not provided except for two studies which included adults with normal cognitive functioning who were frail and needed assistance with two or more ADLs^23^ or had high levels of dependency, including dementia.^27^ The average score on the quality assessment instrument was 14 out of the maximum score of 21 (see Supplementary data Table 2). 

The pooled prevalence estimates for psychological, physical, sexual, and financial abuse and neglect were independently calculated from studies that collected data from older adults and their proxies (Table 1). Visual inspection of the funnel plots indicated that there was evidence of publication bias for physical abuse. Tests of heterogeneity for each of the abuse subtypes were performed. Generally, the studies for each subtype were heterogeneous suggesting that differences in the effect sizes do exist within this set of studies. The Higgins’ *I*^2^ showed high variances for each abuse subtypes (91.1–98.3%) indicating that variance came from sources other than sampling error.

**Figure 2:**
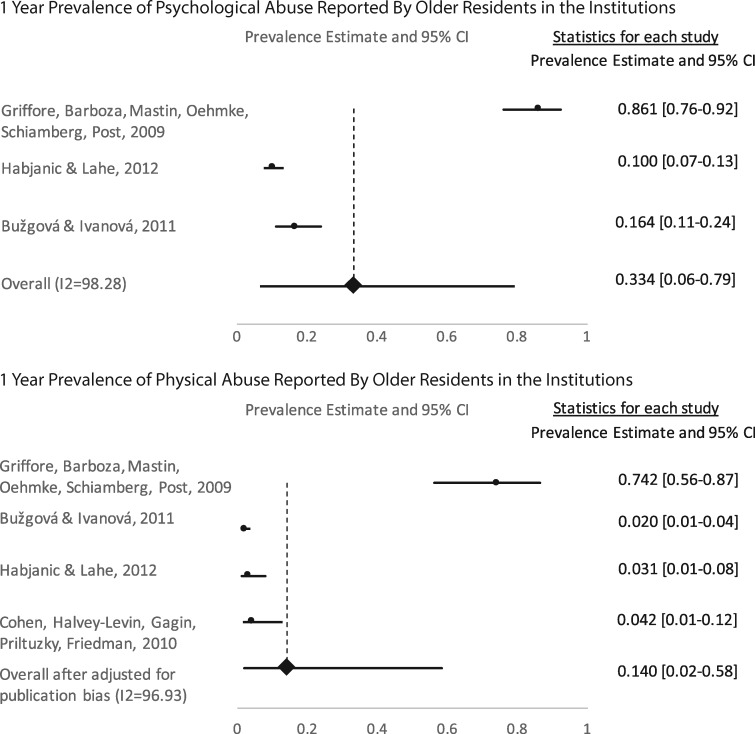

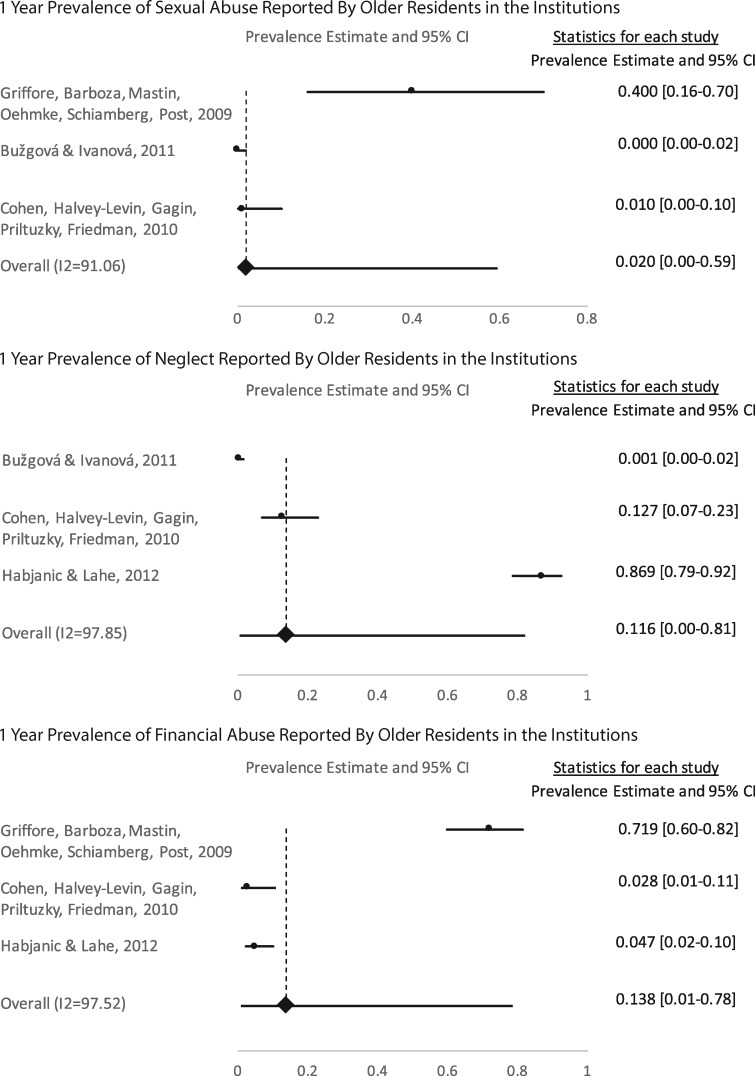
Forest plots of elder abuse subtypes reported by older adults

The rate of psychological abuse was reported in three studies that included a total of 694 individuals. The prevalence estimate for psychological abuse (*Q*[2] = 116.56; *P *< 0.0001; I^2^ = 98.3%) in the past year was 33.4% (CI 6.3–78.9%). There were four studies (*N* = 718) reporting on physical abuse. After adjusting for publication bias, the pooled estimate for physical abuse (*Q*[3] = 97.82; *P *< 0.0001; *I*^2^ = 96.9%) was 14.1% (CI 1.9–58.3%). Sexual abuse (*Q*[2] = 22.38; *P *< 0.0001; *I*^2^ = 91.1%) was reported in three studies (*N* = 569) with a pooled estimate of 1.9% (CI 0.03–59.2%). Financial abuse (*Q*[2] = 80.69; *P *< 0.0001; *I*^2^ = 97.5%) was reported in three studies (*N* = 263) with a pooled estimate of 13.8% (CI 0.7–78.3%). Neglect (*Q*[2] = 92.88; *P *< 0.0001; *I*^2^ = 97.8%) was reported in three studies (*N* = 658) with a pooled estimate of 11.6% (CI 0.4–81.8%). Figure 2 shows the forest plots of the pooled estimates of elder abuse reported by older adults.

**Figure 3:**
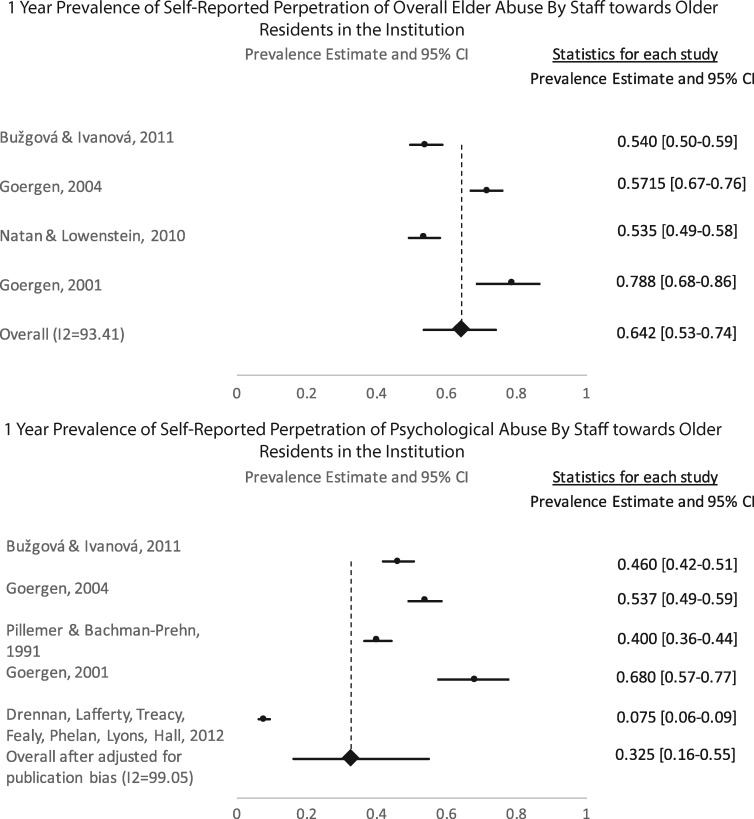

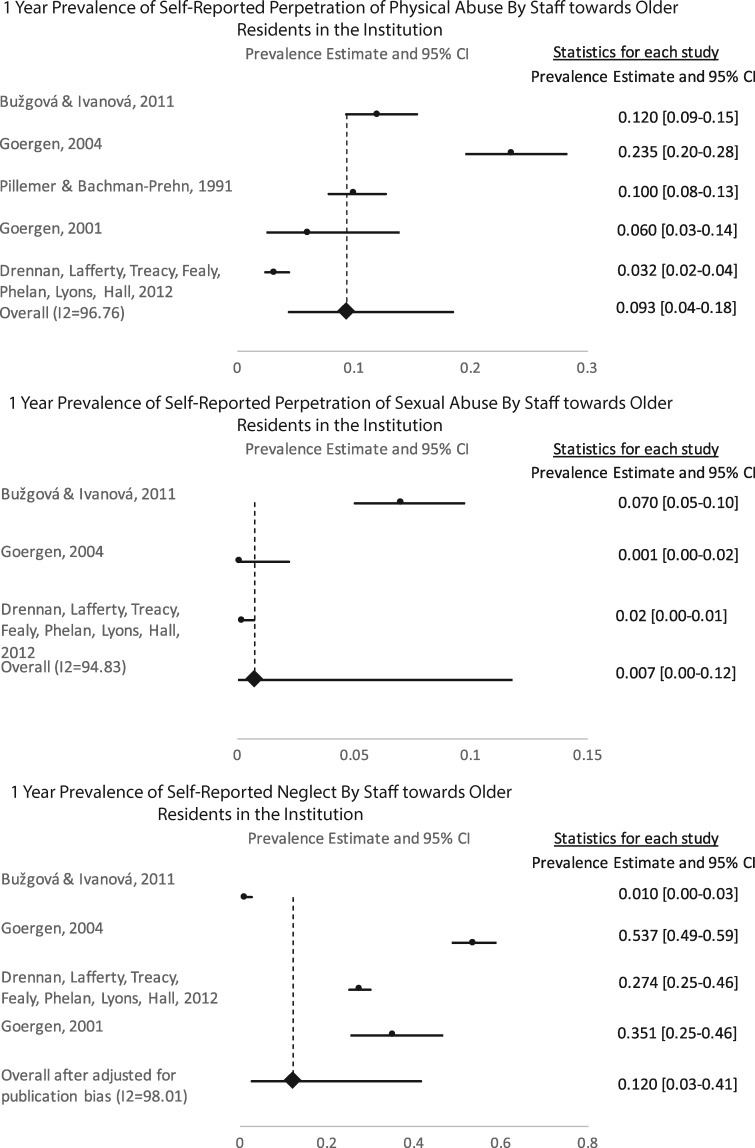
Forest plots of elder abuse subtypes reported by staff

Estimates of perpetrating abuse were calculated from data using staff self-reports for overall abuse as well as abuse subtypes (See Table 1). Evidence of publication bias was present for psychological abuse and neglect. Tests of heterogeneity also revealed a high degree of variance for each abuse subtypes (*I*^2^ = 90–99.1%). There were four studies that provided data for overall abuse (*N* = 1405) with a pooled estimate (*Q*[3] = 45.54; *P *< 0.0001; *I*^2^ = 93.4%) of 64.2% (CI 53.3–73.9%) within the past year. After adjusting for publication bias, the pooled psychological abuse (*Q*[4] = 422.83; *P *< 0.0001; *I*^2^ = 99.1%) rate was 32.5% (CI 16.1–54.6%) and neglect (*Q*[3]=151.04; *P *< 0.0001; *I*^2^ = 98.0%) was 12.0% (CI 2.6–41.4%). There were five studies (*N* = 2706) for psychological abuse and four studies (*N* = 2106) for neglect. The pooled estimate for physical abuse (*Q*[4]=123.47; *P *< 0.0001; *I*^2^ = 96.8%) was 9.3% (CI 4.4–18.4%) with a total of five studies (*N* = 2711). Finally, for sexual abuse (*Q*[2] = 38.72.82; *P *< 0.0001; *I*^2^ = 94.8%), there were three studies (*N* = 2054) with a pooled estimate of 0.7% (CI 2.6–41.4%). Figure 3 shows the forest plots of the pooled estimates of elder abuse reported by the staff.

## Discussion

This is the first rigorous quantitative synthesis of prevalence estimates for elder abuse in the institutions. Findings from this study, based on self report by older residents, show that the past year prevalence of elder abuse in the institutional settings is high. In addition, data based on staff self report, indicate that 64.2% of staff admitted to elder abuse. However, caution is needed when interpreting the estimates from staff self-report. The rates of elder abuse and neglect perpetrated by staff only provide a partial picture on the extent of the problem and do not indicate the overall prevalence of abuse in the institution. Yet, findings from this study is consistent with the anecdotal evidence and the belief that abuse in seniors' residential facilities is widespread.^11^^,^^32^

To date there have been few studies on the prevalence of elder abuse in institutional care settings. Existing studies have provided a wide range of estimates. For example, in studies based on self-reports by older adults or their proxies prevalence estimates have ranged from 31% in Israel for overall abuse^24^—86.9% for neglect in the USA.^26^ Similarly, studies based on staff reports in Germany also indicated a wide range of estimates: from 53.7% for psychological abuse and neglect^28^—78.8% for overall abuse.^29^

This systematic review, based on a comprehensive search strategy, was conducted to better understand the prevalence of elder abuse in institutional settings. Nine studies were synthesized using meta-analysis to pool prevalence estimates for elder abuse. Separate meta-analyses were performed for estimates based on self-reported data by the older adults (i.e. the victims) or their proxies and by the staff (i.e. the abusers). Based on self-reported studies by the staff, 64.2% of them admitted to abuse. Since a minimum of three studies is required to conduct a meta-analysis,^20^ there were not enough studies to be pooled for overall abuse as reported by older residents.

The findings of this study on the self-reported prevalence estimates of elder abuse subtypes by older residents and staff suggest similarities in the magnitude of the problem. The prevalence estimates reported by older residents were highest for psychological abuse (33.4%), followed by physical (14.1%), financial (13.8%), neglect (11.6%), and sexual abuse (1.9%). These rates were higher compared to the prevalence rates in the community settings as reported by older adults: psychological (11.6%), physical (2.6%), financial (6.8%), neglect (4.2%), and sexual (0.9%) abuse.^1^

An examination of risk factors for elder abuse suggests a number of possible explanations for the higher prevalence rate in institutional settings. Although no single risk factors can fully account for the occurrence of elder abuse and research on risk factors in this area suffers from several weaknesses (e.g. lack of unified operational definitions of abuse, measurement problems and inconsistent research methodologies^6^^,^^32^), some risk factors have nevertheless been consistently identified, including the characteristics of victims and staff, of the facilities and the working environment.

The main risk factors for victims of elder abuse are being female, presence of a cognitive impairment and disability, and being older than 74 years old.^33–35^ Research on elder abuse occurring in the community found that the majority of the victims were women. Likewise, 83% of the sample that were included in this meta-analysis was women.^25^ In fact women comprised up to 77.3% of the victims who reported psychological, physical and financial abuse.^25^ The greater share of women in institutional care is consistent with the statistical profile of long-term care facilities in North America and where findings showed that nearly four out of the five residents in care homes are women. This predominance of women stems, in part, from the large differences in gender ratios, especially for the highest age groups.^36^

There is a strong association between increasing dependency and elder abuse occurring in both community and institutional settings.^35^^,^^37^ The risk of dependency also increases with age. The majority of the sample included in the meta-analysis was 75 years and older. Moreover, increased risk for abuse has been associated with declining health in Ireland^38^ and with those needing help with ADLs in Germany.^29^ Such findings are consistent with the sample characteristics included in the meta-analysis where victims of abuse in institutional settings reported frailer health and greater dependency on the staff for assistance in ADLs than non-victims. Of the studies based on self-reports by staff, a small sample of the older residents was diagnosed with dementia. In fact, between 3.4 and 18.5% of the residents who have been abused by staff had dementia.^27^ Older residents in the institutions had many of the risk factors associated with abuse. Such risk factors may also be compounded by the environment in which they lived in.

Nursing homes and other seniors’ residential facilities can be stressful environments. When asked about the main stressors, staff attributed their experience of stress to staff shortages and time pressure.^29^ Indeed research has found that staff who self-reported committing abuse described themselves as emotionally exhausted.^27^^,^^29^ In addition, significant correlation was found between abuse and high ratio of residents to registered nurses.^28^ It was further found that an increased presence of qualified nurses was associated with a reduction in resident abuse risk.^28^ There was wide variation in staff professional experience and training in this meta-analysis. Specifically, in one study, only 48% of the staff were qualified nurses in the field of elder care or medical care^29^ and, in another study, only 10% of the staff were college graduates.^31^

This systematic review has several notable strengths. It is the first of its kind to use meta-analysis to synthesize global prevalence estimates and abuse subtypes in institutional settings based on a comprehensive search strategy. This strategy included studies published in various languages as well as in six different countries. In addition, 26 experts were consulted to further identify any relevant studies that may have been missed in the searches. This review also provided rigorous analyses to compare prevalence rates based on reports by staff and by older adults.

Nonetheless, the findings must be considered in light of several limitations. Prevalence studies were sparse or missing in many regions of the world with a majority of the studies from high-income countries. Furthermore, among the existing studies there was wide variation in methodologies used to measure abuse. The quality of the studies included in the synthesis was poor as reflected in the low average score on the modified Newcastle-Ottawa scale. Due to the sparseness of available literature a more flexible approach had to be adopted with regard to prevalence time periods. One study had a prevalence period of the past 6 months^25^ while the others had prevalence periods of 12 months. Ideally all studies should cover the same time period. Likewise, although most studies included in the meta-analyses were based on self-reports either by older residents or staff, data from one study were based on proxy reports.^26^ Prior studies have indicated that proxy reports might be better at detecting abuse.^39^ Moreover, while efforts have been made to ensure homogeneity of the study samples and to exclude studies with residents with dementia, a small proportion of the samples included residents with dementia.

Given the scarcity of literature, future research should focus on examining elder abuse in institutional settings. In particular, it should clearly define the populations; the types of abuse, such as staff-to-resident abuse, resident-to-resident abuse or visitor-to-resident abuse; characteristics of institutions such as staff to patient ratios, ratio of trained staff, training for staff, and care guidance and adopt a rigorous research methodology particularly in relation to the sampling procedure, use of standardized measurement tools, and method of data collection such as face-to-face interview for older adults and self-administrated questionnaires for the staff. An emphasis on more uniform and systematic quality management strategies for care might result in regular and more systematic administrative data that can be used for future research. The present study found significant heterogeneity for each abuse subtype suggesting that the variance came from sources other than sampling error. Future research is needed to examine these sources of variance by investigating the differences in research methodology that measure and assess institutional elder abuse. Moreover, older people with dementia deserve special attention in future research.

This study makes the following contributions to the field: (i) it provides estimates of abuse as reported by the victims and abusers based on a meta-analysis of all studies showing that this is a large public health problem and (ii) it provides insights into the measurement of elder abuse. Given the similarity in the magnitude of abuse as self-reported by older residents and staff, future data collection can refine reliability and recall issue of abuse by using both older residents and staff within the same institutions. In doing so, it can allow comparability in the prevalence of abuse.

Elder abuse has serious health, social and economic consequences for the victims, their families and the larger societies.^33^ It has been proposed that prevention is more cost-effective than dealing with the consequences of abuse.^16^^,^^33^ The findings of this study have important implications for the quality of care for older adults living in the institutions to ensure that they live without abuse. Both the WHO global strategy and action plan on ageing and health (2016–20) and the WHO strategy and action plan for healthy ageing in Europe (2012–20) affirm the rights of older persons to live with dignity.^15^^,^^16^ These strategies call for strengthening of health and long-term care systems to ensure quality person-centred and integrated care that allows older adults to enjoy their basic human rights and fundamental freedoms.^15^^,^^16^

Crucial to improving the quality of care, there is a need to build capacity of multidisciplinary professionals through training and exchange of good practices across sectors for the prevention of elder abuse. The quality of services requires improvement, in particular through better adaptation to the special needs of older people with functional limitations and by following guidance to prevent elder abuse.^16^

Given that the implementation of quality of care guidelines in long-term care settings is still emerging in many countries, the strategy calls for incorporation of the latest evidence of good practice into national policies and programming to prevent elder abuse. Moreover, these strategies should address negative attitudinal change to avert prejudices towards ageing and to reinforce older people’s fundamental right to live without abuse and violence. There is a need to improve the evidence based on sound models of care and to strengthen research capacity on effective preventive interventions.^16^

An OECD report showed that while most countries have several mechanisms to address abuse such as legislation to encourage public disclosure of specific cases; provision of complaint mechanisms and establishment of ombudsman, few countries have been systematically measuring whether long-term care is safe, effective and meets the needs of care recipients.^40^ The findings of this study emphasize the urgency of the demand for better, higher-quality care of older adults. This is particularly relevant given the demographic challenge of ageing societies in middle- and high-income.

Despite higher rates of abuse and neglect in the institutional settings than in the community settings, elder abuse in the institutions has not achieved the same public health priority as other forms of abuse. Greater attention and resources are needed to ensure that nursing and residential home facilities strike a balance between providing care for the complex needs of older residents and ensuring proper support of the staff through training, education and adequate manpower and wages to ensure quality of care. Given the rapid ageing of the population, the findings of this study strengthen the case for global action to expand efforts in researching, preventing and supporting victims of institutional abuse. Investment in developing interventions for older adults and the staff in institutional facilities must be a public health priority to help reduce the effect of elder abuse.

## Supplementary Material

Supplementary Data
