# Supplementary material for: The prevalence of elder abuse in institutional settings: a systematic review and meta-analysis
Source: Eur J Public Health. 2018 Jun 5;29(1):58–67. doi: 10.1093/eurpub/cky093 (PMC6359898; doi:10.1093/eurpub/cky093)
Supplement: Supplementary Data [file cky093_supplementary_data.docx]

**Supplementary Table: Characteristics of prevalence studies included in meta-analysis for elder abuse in the institution**
